# Reconceptualization of Hormetic Responses in the Frame of Redox Toxicology

**DOI:** 10.3390/ijms23010049

**Published:** 2021-12-21

**Authors:** Zoi Skaperda, Fotios Tekos, Periklis Vardakas, Charitini Nepka, Demetrios Kouretas

**Affiliations:** 1 Laboratory of Animal Physiology, Department of Biochemistry-Biotechnology, School of Health Sciences, University of Thessaly, 41500 Larissa, Greece; zoskaper@bio.uth.gr (Z.S.); ftekos@uth.gr (F.T.); periklis_vardakas94@hotmail.com (P.V.); 2 Department of Pathology, University Hospital of Larissa, 41334 Larissa, Greece; cnepka@yahoo.gr

**Keywords:** hormesis, oxidative stress, polyphenols, redox biomarkers, dose, duration of exposure

## Abstract

Cellular adaptive mechanisms emerging after exposure to low levels of toxic agents or stressful stimuli comprise an important biological feature that has gained considerable scientific interest. Investigations of low-dose exposures to diverse chemical compounds signify the non-linear mode of action in the exposed cell or organism at such dose levels in contrast to the classic detrimental effects induced at higher ones, a phenomenon usually referred to as hormesis. The resulting phenotype is a beneficial effect that tests our physiology within the limits of our homeostatic adaptations. Therefore, doses below the region of adverse responses are of particular interest and are specified as the hormetic gain zone. The manifestation of redox adaptations aiming to prevent from disturbances of redox homeostasis represent an area of particular interest in hormetic responses, observed after exposure not only to stressors but also to compounds of natural origin, such as phytochemicals. Findings from previous studies on several agents demonstrate the heterogeneity of the specific zone in terms of the molecular events occurring. Major factors deeply involved in these biphasic phenomena are the bioactive compound per se, the dose level, the duration of exposure, the cell, tissue or even organ exposed to and, of course, the biomarker examined. In the end, the molecular fate is a complex toxicological event, based on beneficial and detrimental effects, which, however, are poorly understood to date.

## 1. Introduction

For several decades there was a general belief that the effects arising from exposure to diverse doses of xenobiotics follow a linear pattern, resulting in an enormous knowledge gap regarding the low-dose zone responses [1]. Nevertheless, in the recent past, numerous studies have revealed an inverse response not only to various doses of foreign chemical substances and stressors, but also of bioactive compounds, overturning the notion of linearity and the threshold-response models of dose determination [2,3]. This phenomenon has been described in several studies as “biphasic dose response” and has emerged as a critical step in establishing the modality of each compound [4,5,6,7]. In fact, the adoption of the specific term could determine the optimal dose level of a chemopreventive agent, thus advocating Philip von Hohenheim’s adage “Die Dosis macht das Gift” or “dose makes the poison” [8]. 

The concept of “hormesis” was firstly introduced in 1943 to describe how low doses of red cider tree extracts enhanced fungi proliferation in a biphasic manner [9]. From then on, many researchers have investigated not only if these low-dose effects represent a common biological feature, but also the main contributors implicated in such outcomes. As a result, biphasic responses have been reported by researchers assessing the chemopreventive properties of several phytochemical compounds and food components [10,11]. In the scientific fields of Biology and Medicine, the definition of hormesis has been proposed as the adaptive response of both cells and organisms to a specific level of a stressful stimuli, e.g., exercise, fasting, and exposure to low doses of xenobiotics. In other words, hormesis can be defined as the phenomenon wherein a harmful substance or a stressful condition exerts both stimulating and beneficial effects to living organisms, in those cases where the quantity or the intensity of a harmful substance or stimuli, respectively, is extremely low [12,13].

A controversial issue that has gained considerable interest in recent years is radiation hormesis [14]. According to this, low doses of ionizing radiation, within the region of and just above the natural background levels, might stimulate the activation of distinct repair mechanisms, that are cytoprotective and would not be activated in the absence of the respective stimuli [15]. In support of this argument, toxicological and epidemiological investigations conducted to evaluate the effects of low levels of ionizing radiation report the induction of useful cellular adaptive mechanisms [16]. Contrariwise, the linear no-threshold model (LNT) of radiation, the cornerstone of the existing regulatory frameworks, stresses that even low levels of ionizing radiation comprise a potential threat for carcinogenesis. Although the specific model has been adopted by various organizations to set their protection policies and regulatory standards, it is latterly contested [17,18]. As a result, to date, it remains still unclear whether exposure to such radiation levels constitutes a risk factor in the long run or exerts a beneficial effect. In contrast, high-intensity exercise represents a well-established condition associated with adaptive mechanisms. To be more specific, a challenging weights workout promotes, for a certain period of time, oxidative stress and inflammatory responses, causing a relatively low and manageable muscle injury [19]. The human organism responds to this microtrauma by repairing the slightly damaged muscle fibers or creating new ones and, consequently, exercise improves muscles’ strength capacity. Furthermore, enzymes of cytochrome P450 (CYPs) and glutathione S-transferases, both constituting the metabolic system responsible for the detoxification and excretion of xenobiotics, are also prone to the induction of hormetic responses [20]. Mithridatism, a term derived from the “Poison King”, Mithridatis VI Eupator, refers to poison resistance acquired through the long-term exposure to extremely low levels of toxic agents that enhance the activation of metabolizing enzymes or change the metabolic pattern [21]. Indeed, low and nontoxic levels of a broad range of chemical substances and drugs, but also phytochemicals, coordinate the upregulation of metabolizing enzymes, an effect associated with the prevention from molecular damage and, as a result, from the onset of several pathologies, among which cancer [22]. Nevertheless, elevated exposures to specific pharmaceuticals and phytochemicals are able to selectively inactivate CYPs, endangering the survival of the corresponding biological system [23]. 

**Figure 1:**
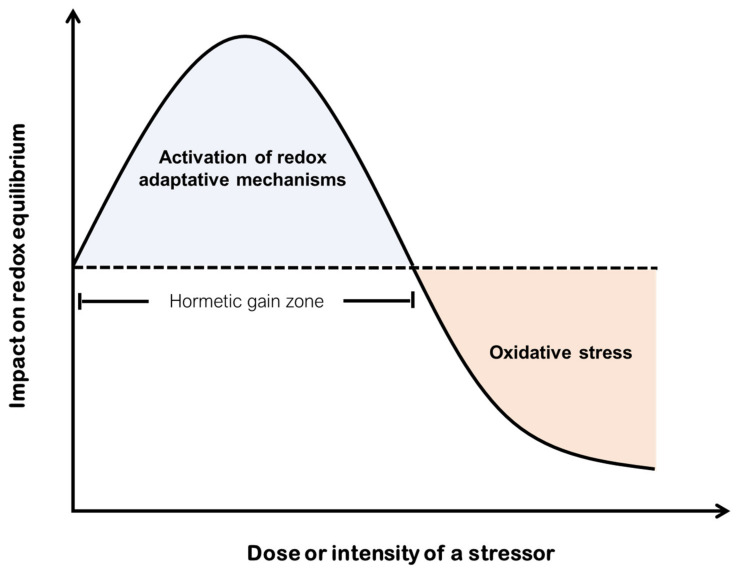
Illustrative depiction of hormetic responses following exposure to a redox altering stimulus. Exposure to low doses or intensity of a stressor triggers the upregulation of antioxidant defense mechanisms to prevent from oxidative damage. In contrast, high levels of a stressor disrupt redox balance resulting in toxicity via the emergence of oxidative stress and potentially in cell death.

In the present review we focus on a specific aspect of hormesis and, in particular, on the ability of cells to adapt to a changing redox potential, called redox regulation. Oxidative stress, a crucial parameter in modes of hormetic responses, is considered a single toxicodynamic endpoint (Figure 1). However, the redox biomarkers evaluated to demonstrate its gradation levels in vitro and in vivo are numerous and depend on multiple factors [24]. Besides the variability in redox biomarkers, as a part of the determination of hormetic responses, dose levels, exposure time, as well as the different biological systems examined, constitute crucial modulators of hormesis. Due to the fact that the existing literature is very heterogeneous, it is rather a difficult task to draw general conclusions regarding the significance of redox biomarkers and the beneficial or harmful effects originating from their upregulation or downregulation. Hence, the hormesis modulators should be carefully taken into account. Phytochemicals are the most important dietary factors directing the regulation of redox responses. As such, analytical issues surrounding their biphasic effects on redox biomarkers have received substantial attention as humans consume polyphenol-rich foods in a lifetime [25]. 

**Figure 2:**
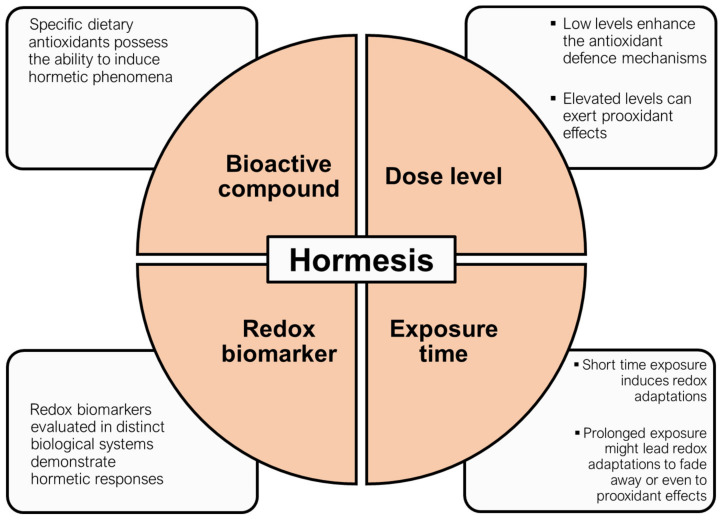
The four pillars of hormetic phenomena from a redox aspect.

Collectively, the aim of this review is to summarize the results of our extensive research in a vast number of bioactive compounds, that exert antioxidant properties, consisting of plant extracts, food components, beverages, and dietary supplements, in doses below the region of adverse responses or threshold dose, which are of particular interest and are characterized as the “hormetic gain zone”. In this way, we demonstrate that practically this zone depends on many parameters and is not something homogenous in terms of the biological fingerprint that leaves to the human body. In addition, our reconceptualization proposes specific endpoints that should be carefully considered when analyzing results that describe the effects of foods, beverages, and extracts with antioxidant properties on a biological system (Figure 2). 

## 2. Hormetic Effects of Diverse Bioactive Compounds

Induction of hormesis has been documented after exposure to a broad range of natural and chemical agents. Nevertheless, to date, it is not yet fully elucidated whether the provocation of biphasic dose responses is the result of a single molecular mechanism. Several studies have indicated the antioxidant or prooxidant effects of individual foods, herbs, dietary supplements, coffee extracts, olive oil extracts, polyphenolic compounds, and specific phytochemicals. Of note, the exerted harmful or beneficial effects strongly depend on the administered agent and its mode of action. 

In a previous study of our research group, the antioxidant properties of olive oil polyphenol extracts, with substantial differences in terms of their hydroxytyrosol and tyrosol content, were evaluated, revealing biological activity of utmost importance in human cervical adenocarcinoma HeLa cells [26]. More elaborately, the hydroxytyrosol-rich extract increased the intracellular levels of reduced form of glutathione (GSH), an important antioxidant molecule [27], at doses of 15.0 and 20.0 μg of extract, whereas the tyrosol-rich extract increased GSH levels in a wider dose range (10.0, 15.0, 20.0, and 25.0 μg of extract). Additionally, GSH levels were elevated after the administration of olive oil extracts containing an equal amount of hydroxytyrosol and tyrosol. These extracts caused an increase in GSH levels at 2.5, 5.0, 10.0, and 25.0 μg of extract. Conclusively, tyrosol-rich extracts were more efficacious in terms of improving the cellular antioxidant profile as compared to the hydroxytyrosol-rich extracts, an assertion supported by the lower concentrations required for increasing GSH levels [26].

In contrast, the assessment of the antioxidant properties of a total phenolic fraction, derived from an extra virgin Greek olive oil, demonstrated a different mode of action. In particular, the administration of the extract in human hepatocarcinoma HepG2 cells enhanced the intracellular GSH levels at the dose of 1 μg/mL, whereas at the dose of 1.5 μg/mL GSH levels were decreased, implying a prooxidant effect of the specific fraction in higher concentrations. Furthermore, the same sample decreased TBARS levels, a well-established biomarker of lipid peroxidation, in doses lower than 7.5 μg/mL, but exerted an opposite effect in higher doses when administered in human endothelial EA.hy926 cells [28]. 

Sulforaphane, a sulfur-rich phytochemical compound routinely encountered in cruciferous vegetables, elicits biphasic effects when administered in human mesenchymal stem cells. Administration of sulforaphane at a low dose, i.e., 1 μΜ protects cells from oxidative modifications of macromolecules and apoptotic cell death, whereas at a higher dose, i.e., 20 μM, oxidative DNA damage and cell death are promoted [29]. Obviously, the observed hormetic responses are a combinatorial result, emerging from the nature of the specific compound and the administered dose. Another phytochemical compound, namely quercetin, also possesses the capacity to exhibit such biphasic effects. Doses ranging from 1 to 40 μM exhibit antioxidant properties, whereas doses exceeding 40 μM are correlated with disturbances of redox equilibrium and cytotoxicity [30]. 

In a recent study, the individual administration of vitamin C in gastric cancer MKN45 cells and HeLa cells showed an increase in intracellular GSH levels, while the administration of dietary supplements, containing not only vitamin C, but also a series of phytochemical compounds with significant chemoactive and bioactive properties, induced a depletion of GSH levels. The aforementioned study designates the ability of the compounds comprising three novel dietary supplements to exhibit a synergistic effect, thus overcoming the capacity of the equivalent quantity of pure vitamin C and indicates a distinct mode of action depending on each compound. Hence, specific compounds are more potent than others in inducing hormetic responses via a specific pathway, revealing the importance of the compound’s or mixture’s reactivity factor [31]. 

## 3. Dose Is a Decisive Factor for the Manifestation of Biphasic Effects

A major point for achieving successful and targeted interventions is the selection of the appropriate dose. Interindividual variability is influenced by several parameters among which gender, age, health status, diet, exercise, and genetic factors. Moreover, environmental factors, such as exposure to xenobiotics, as well as endogenous factors, such as circadian rhythms, might also affect the selection of the proper dose. For instance, exercise ameliorates the symptoms of type 2 diabetes mellitus and leads to elevated mitochondrial biogenesis. As a result, there is an accumulation of reactive oxygen species (ROS), as manifested by the increased biomarkers of oxidative modifications which, afterward, return to basal levels [32]. Consistent with the concept of mitohormesis, exercise-induced oxidative stress ameliorates insulin resistance, causing a graded adaptive response that enhances the antioxidant defense mechanisms [33]. Importantly, supplementation with antioxidant-containing mixtures has been reported for blunting these health-promoting effects of exercise in humans [34].

The hormetic dose response may result either from a direct stimulation or as a compensatory mechanism after the disruption of redox homeostasis [35]. Therefore, the quantitative characteristics of hormetic dose responses appear to be similar regardless of the mode of action by which the stimulation occurs. More specifically, biphasic dose responses confer that at high doses, within a toxicological condition, the typical endpoints measured indicate the provocation of cellular damage. Nevertheless, when the dose decreases below the threshold, the low-dose stimulation leads to an adaptive response, that conforms to a measure of biological bioactivity, as denoted in the cases of upregulation of GSH and downregulation of ROS, as well as other biomedical endpoints of interest [36]. 

Previously, Kouka et al. examined the antioxidant activities of a total polyphenolic fraction and individual hydroxytyrosol from olive oil derived from a Greek *Olea europaea* variety in EA.hy926 cells and mouse myoblast C2C12 cells. At the highest administered dose, both the polyphenolic fraction (10 μg/mL for C2C12 cells and EA.hy926 cells) and hydroxytyrosol (25 μg/mL for C2C12 cells and 1 μg/mL for EA.hy926 cells) increased GSH levels, however to a minor extent than at lower concentrations. A potential prooxidant activity of polyphenols after reaching a critical concentration might be responsible for the decreased potency of the two samples examined, illustrating their dose dependent biphasic effects [37]. In line with olive oil compounds, polyphenolic extracts from olive tree blossoms exerted hormetic responses when examined in in vitro cell-based systems. Regarding the effects on HepG2 cells, the olive tree blossom extract increased GSH levels at doses of 0.5 to 2.5 μg of extract as compared to the control group. Nevertheless, a prooxidant effect was observed when the dose reached 5 μg of extract, as depicted by the decreased GSH levels. Additionally, ROS levels were decreased after administration of 0.5, 1, 2.5, and 5 μg of the same sample. Moreover, GSH levels were increased after the administration of 40 and 60 μg of an olive tree blossom extract derived from the variety Lianolia from Corfu Island. However, as previously observed when the dose reached the crucial levels of 80 μg of extract, GSH levels were decreased, demonstrating a prooxidant effect [26].

Dose-dependent hοrmetic effects, related to redox processes, were also observed in the case of green and roasted coffee bean extracts administration. In brief, supplementation of the coffee extracts in C2C12 cells resulted in elevated GSH levels in the dose range of 100–800 μg/mL. A finding of particular interest was the non-linear elevation in GSH levels as an excessive increase was observed at 400 µg/mL, followed by a subsequent decline, whereas no effect was observed at 1600 µg/mL [38]. As previously reported, a prooxidant effect after reaching a specific dose is a common phenomenon, also emerging after administration of other plant polyphenolic extracts. 

Curcumin, a bioactive polyphenolic compound, exerted hormetic responses in human skin fibroblasts ASF-2 in a dose-dependent mode. In particular, administration of low doses of curcumin, i.e., below 20 μM, fortified the redox milieu, as denoted by the intensification of antioxidant enzymes activity (heme oxygenase 1, glutathione-S-transferase) and antioxidant molecules levels (GSH). On the other hand, treatment with 30 μM of curcumin impaired redox balance, an assertion supported by the elevation in ROS levels and the depletion of GSH levels, thus promoting necrotic cell death [39]. 

Vitamin C, a water-soluble vitamin with major antioxidant properties, exhibited biphasic dose responses when supplemented after the induction of hepatic ischemia/reperfusion in a rat model. In brief, the intravenous administration of vitamin C at low doses, i.e., 30 mg/kg body weight and 100 mg/kg body weight, attenuated lipid peroxidation in the liver after 1h and 5h, as compared to the ischemia/reperfusion group. Contrariwise, the highest administered dose of vitamin C, i.e., 1000 mg/kg body weight, adversely affected liver redox status, as depicted by the advanced lipid peroxidation in comparison with the ischemia/reperfusion group after 5h, thus exerting prooxidant effects [40].

Melatonin, a hormone synthesized and secreted by pineal gland [41], but also received to a lesser extent from dietary sources [42], has been shown to be an efficacious free radical scavenger, thus attenuating the detrimental effects of oxidative stress [43]. In a previous study conducted to assess the ameliorating effects of melatonin on oxidative stress in an Alzheimer’s disease model, biphasic dose responses were observed in terms of redox status. To be more specific, brain slices obtained from a mouse model were treated with amyloid β-protein (Aβ) (25 μM and 50 μM) to induce oxidative stress, melatonin (100 μM and 1 mM) or a combination of them (50 μΜ Aβ—100 μΜ melatonin). Interestingly, although melatonin administration at a low dose was adequate to prevent from Aβ-induced oxidative stress, the administration of the highest dose individually, i.e., 1 Mm, elevated significantly redox active iron levels and heme oxygenase-1 (HO-1) immunoreactivity, indicating that melatonin supplementation at millimolar levels might exert prooxidant effects [44].

Cellular adaptive mechanisms activated to prevent from mortality under the influence of stressors include, among others, the upregulation of vitagenes, major genes that encode molecules with antioxidant and cell-protective properties [45]. Oxidative stress as a harmful condition challenging cellular homeostasis promotes the expression of vitagenes, producing members of heat shock proteins (Hsps) family, such as Hsp32, else known as HO-1 and Hsp70 [46]. In general, Hsps play a pivotal role in protein folding, in preventing from protein aggregation, and in protein degradation [47]. As a result, the upregulation of specific family members is often observed in response to a redox altering stimulus to ensure cell survival. For instance, that was the case for sodium arsenite that induced biphasic effects on human embryo lung fibroblast HELF cells in a dose-dependent manner. To be more specific, low doses of sodium arsenite, i.e., 0.1 μM and 0.5 μM, enhanced cell proliferation, whereas exposure to higher doses, i.e., 5 μM and 10 μM, inhibited cell growth. In terms of redox adaptations, superoxide dismutase (SOD) activity was elevated at low doses (0.5 μM) and decreased at higher ones (5 μΜ and 10 μΜ), while a significant effect was also observed in the expression of heat shock protein 27 (Hsp27), a chaperone protecting from the pernicious effects of oxidative stress; at early exposure times and low doses Hsp27 expression was elevated, followed by decrease at prolonged exposure times and high doses [48]. 

Except for redox regulation, hormetic effects are also observed after evaluating other critical endpoints. Previously, the effects of long-term, low-dose exposures to a chemical mixture comprising six pesticides were evaluated on rat redox status. The results highlighted the association of extremely low doses with vitamin deficiency and with neurobehavioral alterations of the experimental animals, as manifested by specific effects on central nervous system [49]. The same experimental protocol was used to assess alterations on locomotor activity and changes in behavioral status. According to the results, the rats exposed to the pesticide mixture exhibited a dose-dependent stimulation of the nervous system [50].

## 4. The Role of Exposure Time in the Emergence of Hormetic Responses

Time is a crucial parameter for targeted interventions, aiming to prevent from diseases by successfully coping up with alterations in the cellular environment, e.g., oxidative stress [51]. In cases of elevated levels of oxidative stress in cells, flavonoids can act as direct scavengers of the detrimental ROS. Subsequently, they are oxidized producing a quinone that selectively reacts with thiols, such as GSH, thus enhancing the endogenous antioxidant system. When the oxidative stress levels continue to increase, thiol-containing proteins are also targeted; concerning flavonoids, this could be Keap1 [52]. Potential outcomes could be the increased expression of Nrf2-targeted antioxidant genes. Nevertheless, when oxidative stress levels reach a higher threshold as well as last for a long period of time, even more thiol-containing proteins are attacked. For instance, Ca^2+^-ATPase could selectively create a hazardous cellular environment by inducing toxicity and apoptotic cell death [53,54]. Therefore, the duration of exposure or even the right timing between the first and the second exposure are critical parameters. In case that the time interval between the first and the second exposure is quite short, time is not sufficient for the induction of a protective adaptive response and the second exposure might be lethal. On the contrary, if this time period is elongated, the adaptive effect could fade away and, as a result, the first treatment exhibits no effect. 

Humans are daily exposed to complex mixtures of xenobiotics with the low-dose regimen. Fountoucidou et al. demonstrated that a 12-month exposure to three dosage levels of a complex chemical mixture, containing pesticides, food additives, and preservatives, well below the regulatory limits (0.0025 × NOAEL, 0.01 × NOAEL, and 0.05 × NOAEL), induced useful adaptations in blood’s redox state (i.e., increased GSH, catalase and total antioxidant capacity and decreased protein carbonyls and TBARS). Contrariwise, exposure to the highest dose for 18 months induced significant perturbations in blood and tissue redox profile (i.e., increased protein carbonyls and TBARS). This study simulates a scenario of real-life risk exposure to mixtures of xenobiotics. Low concentrations of reactive species are able to induce adaptive responses, thus protecting blood and tissues from their detrimental impact. On the contrary, the long-term (i.e., 18 months) exposure to the highest dose of the mixture induced significant disturbances of redox equilibrium in blood and in the majority of the tested tissues [55]. 

Unfortunately, most of the epidemiologic and experimental studies in literature have investigated time-dependent effects of single chemical compounds rather than chemical mixtures. Previously, bisphenol A was reported to increase intracellular ROS levels in a time-dependent manner after 12 h of exposure. Nevertheless, it is meaningful to highlight the fact that in a prolonged duration of exposure, i.e., for 18 h and 24 h, ROS levels return to pre-exposure levels [56].

With regards to phytochemicals, treatment of granuloma cells with increasing doses of resveratrol (0.1–50 μM) for 24 h and 120 h triggered a dose- and time-dependent prooxidant effect, as shown by the increased ROS levels. Therefore, the authors demonstrated that the time of exposure and the dose induced increment of ROS production in a dependent manner. When examining other biomarkers, cells treated for 24 h with 1–50 μM of resveratrol exhibited a decrease in SOD activity, whereas all cells treated for 120 h displayed an increase in the specific enzyme activity, thus revealing an interaction between the time of treatment and resveratrol dose. The elevated TBARS levels observed in 24 h were decreased after a prolonged resveratrol treatment for 120 h, showcasing that lipid peroxidation levels were dependent on exposure time [57]. 

Except for single polyphenolic compounds, studies investigating polyphenol-enriched byproducts have had a significant impact. For instance, olive mill wastewater incorporated into broilers’ feed was evaluated for its antioxidant capacity [58]. More specifically, two feeding groups composed by twenty-four broilers received olive oil mill wastewater-enriched or common feed for 37 days. The blood sampling time points were 17, 27, and 37 days and the at the end of the dietary intervention the experimental animals were sacrificed and muscle, heart, and liver were excised and collected. According to the results, GSH levels were statistically increased at 17 days in the experimental group as compared to the control group, whereas at days 27 and 37 GSH levels were decreased in the experimental group, highlighting the prooxidant effect of these polyphenol-enriched feed in the long run. In general, the findings of this study suggest that feed supplemented with olive oil mill wastewater compounds could be useful for enhancing the redox status of broiler chickens by reducing the oxidative damage in fundamental biomacromolecules, such as proteins and lipids and by inducing the activation of antioxidant defense mechanisms, such as catalase activity, GSH, and total antioxidant capacity. Conclusively, the prolonged duration of exposure in the polyphenol contained feed might result in hormetic effects [58]. 

Natural extracts containing various bioactive components have been examined for their intracellular effects, revealing time-dependent hormetic effects. C2C12 cells and EA.hy926 cells were treated with a grape pomace extract at low, non-cytotoxic concentrations for 3, 6, 12, 18, and 24 h. The results demonstrated that the treatment with the grape pomace extract reduced both the expression and the activity of catalase (CAT), an enzymatic mechanism implicated in the decomposition of hydrogen peroxide (H_2_O_2_) in endothelial cells, whereas in mouse myoblasts exerted no effect. Moreover, in C2C12 cells CAT expression was not affected when both concentrations were administered for 3 h. Interestingly, when the duration of exposure to grape pomace extract was 6, 12, 18, and 24 h a time-dependent decrease of CAT expression was observed [59]. 

## 5. Evaluation of Distinct Redox Biomarkers Reveals Biphasic Effects In Vitro and In Vivo

Redox Biology has “adopted” specific and sensitive tools to evaluate the levels of oxidative stress, the so-called redox biomarkers. Due to the fact that the literature is very heterogeneous, it is often confusing to draw general conclusions on the significance of redox biomarkers when assessed in distinct biological systems. Among the main criteria that they should fulfill, of great importance remains their ability to knowingly reflect a biological condition or phenomenon [60]. Oxidative or reductive stress are indisputably common toxicological mechanisms across diverse chemical agents, unifying the biological action of broad classes of physichochemically disparate compounds, without excluding agents of natural origin, such as food components, beverages, phytochemical compounds, and several food additives [61]. Reactive oxygen and nitrogen species, ROS and RNS, respectively, generated after exposure in such agents, are known to elicit detrimental effects on cellular biomolecules, i.e., lipids, carbohydrates, proteins, and nucleic acids. Hence, several antioxidant defense mechanisms are implicated within the cells in order to prevent from redox dysregulation. Emerging research evidence suggests that compounds with antioxidant properties can confine the pernicious outcomes of chemical compounds by interrupting the propagation of free radicals, subsequently restoring redox balance [62]. 

Hormetic mechanisms of polyphenol-containing mixtures have been previously studied for their protective activities against oxidative modifications [63]. The supplementation of grape pomace extract in C2C12 cells and EA.hy926 cells enhanced the antioxidant defense mechanisms both in the presence or absence of an oxidizing agent. More specifically, treatment of C2C12 cells with the grape extract at concentrations of 5 and 10 mg/mL, with prior exposure to tert-Butyl hydroperoxide (t-BHP) significantly reduced ROS levels, as compared to the individual administration of t-BHP. Moreover, the grape extract increased GSH levels at 2.5, 5, and 10 mg/mL in comparison with the t-BHP administration. An important finding obtained in that study was that the highest administered concentration of the grape extract restored GSH to similar levels to t-BHP treatment, presumably indicating a biphasic effect of that extract when examined in higher concentrations [64]. 

Epigallocatechin-3-gallate, a polyphenolic compound found in green tea, has been proposed to upregulate HO-1 expression. The molecular mechanism implicated in this upregulation is the activation of NF-E2-related factor 2 (Nrf2)/antioxidant response element (ARE) pathway, that confers resistance against cell death induced by oxidants, suggesting a hormetic mechanism of action [65]. 

A previous study evaluated the antioxidant potential of a grape pomace-enriched feed administered in piglets. To this end, 24 piglets, 20 days-old, were assigned to two experimental groups receiving standard or experimental diet for 30 days. According to the results, several biomarkers were upregulated or downregulated, depending on the examined tissue, as well as on the duration of feed administration. In particular, total antioxidant capacity (TAC) levels in plasma and brain were decreased after receiving the experimental feed for 35 days, whereas its levels were increased in pancreas and stomach. Moreover, in the same study, H_2_O_2_ decomposition rate was increased when examined in kidneys after 35 days of administration of grape pomace-enriched feed and decreased when examined in lung and stomach [66].

The comparative effects of three dietary supplements in parallel with vitamin C administration in three separate in vitro cell based systems were evaluated to showcase distinct mechanisms of action. The dietary supplements significantly decreased GSH levels and increased ROS and TBARS levels in MKN45 cells at all tested concentrations. On the other hand, vitamin C caused an increase in GSH levels at the three highest non-cytotoxic concentrations (0.015–0.06 mg/mL), revealing that this molecule, in contrast with the dietary supplements, did not possess the ability to suppress the antioxidant mechanisms of the specific cancer cell line. Critical is the fact that vitamin C revealed its chemoprotective action through a different mechanism, as manifested by the increase in ROS levels at the two highest non-cytotoxic concentrations examined (0.03 and 0.06 mg/mL). Regarding TBARS levels, all the examined concentrations of vitamin C (0.0075–0.06 mg/mL) caused advanced lipid peroxidation, as compared to the control. In contrast, when the dietary supplements and vitamin C were examined in HepG2 cells a different pattern was observed. In brief, both the dietary supplements and vitamin C diminished GSH pools and elevated ROS and TBARS levels, revealing the biphasic effects of vitamin C in distinct cellular systems [31]. 

## 6. Concluding Remarks

The present review summarizes biphasic effects that are detected in various biomarkers and depend on the specific compound, dose levels, duration of exposure, and the biological system examined in a number of studies associated with effects on redox homeostasis. The consistency of the vast array of hormetic findings strongly suggests that the observed biphasic responses may be a manifestation of the plasticity of biological systems. A central finding within the biological sciences, which, however, is poorly understood, is the fact that essentially all biological models respond to external stress using similar features for their responses that are dynamically altered with the dose, time, component, and the specific biomarker of exposure. Collectively, numerous physiological processes are subjected to hormetic responses and several factors, that are outside the scope of investigation, are involved in inducing an adaptive response. Until now, hormesis concept seems to attract low general acceptance, due to generally faint stimulatory effects and the lack of mechanistic explanations in all the factors implicated with hormetic phenomena. Better understanding of hormetic responses will provide a useful tool for disease interventions aiming to increase our ability to adapt, as well as prevent disease progression. In this context, in the present report, we demonstrate clear evidence that the hormetic zone is a dynamic balance within the bounds of health and disease and we create the margin between a “good” and “bad” agent on a more holistic fashion.
